# A Comprehensive Assessment of Cardiomyopathies through Cardiovascular Magnetic Resonance: Focus on the Pediatric Population

**DOI:** 10.3390/diagnostics12051022

**Published:** 2022-04-19

**Authors:** Francesca Baessato, Cristina Romeo, Mark G. Rabbat, Gianluca Pontone, Christian Meierhofer

**Affiliations:** 1 Congenital Heart Disease and Pediatric Cardiology, German Heart Center Munich, Technical University of Munich, 80636 Munich, Germany; meierhofer@dhm.mhn.de; 2 Department of Cardiology, Regional Hospital S. Maurizio, 39100 Bolzano, Italy; cristina.romeo@sabes.it; 3 Division of Cardiology, Loyola University Medical Center, Chicago, IL 60153, USA; mrabbat@lumc.edu; 4 Cardiovascular Imaging Department, Centro Cardiologico Monzino IRCCS, 20138 Milan, Italy; gianluca.pontone@cardiologicomonzino.it

**Keywords:** cardiovascular magnetic resonance, hypertrophic cardiomyopathy, dilated cardiomyopathy, left ventricular non compaction, diagnostic accuracy, prognosis

## Abstract

Cardiomyopathies (CMPs) are a heterogeneous group of diseases that involve the myocardium and result in systolic or diastolic impairment of the cardiac muscle, potentially leading to heart failure, malignant arrhythmias, or sudden cardiac death. Occurrence in pediatric age is rare but has been associated with worse outcomes. Non-invasive cardiac imaging techniques, integrated with clinical, genetic, and electrocardiographic data, have shown a pivotal role in the clinical work-up of such diseases by defining structural alterations and assessing potential complications. Above all modalities, cardiovascular magnetic resonance (CMR) has emerged as a powerful tool complementary to echocardiography to confirm diagnosis, provide prognostic information and guide therapeutic strategies secondary to its high spatial and temporal resolution, lack of ionizing radiation, and good reproducibility. Moreover, CMR can provide in vivo tissue characterization of the myocardial tissue aiding the identification of structural pathologic changes such as replacement or diffuse fibrosis, which are predictors of worse outcomes. Large prospective randomized studies are needed for further validation of CMR in the context of childhood CMPs. This review aims to highlight the role of advanced imaging with CMR in CMPs with particular reference to the dilated, hypertrophic and non-compacted phenotypes, which are more commonly seen in children.

## 1. Introduction

Cardiomyopathies (CMPs) are defined as a heterogeneous group of diseases of the myocardium characterized by structural, mechanical, and electrical abnormalities which result in systolic or diastolic dysfunction. These conditions refer to a wide group of etiologies and associated genetic, infectious, metabolic, or neuromuscular conditions [1]. 

In pediatric age, CMPs are rare disorders, with an annual incidence between 0.65 and 1.24 per 100,000 children, which becomes significantly higher in the first 2 years of life [2]. 

In the last few years, several classification systems have evolved due to advances in genetic examinations and evolution of imaging techniques. The most frequent type of CMPs in pediatric age is dilated CMP (DCM), which accounts for about half of the cases, followed by hypertrophic CMP (HCM), which is the second most common group. Rarer CMPs are left ventricular non-compaction (LVNC) and restrictive phenotypes. Of note, arrhythmogenic CMP (ARVC) is rarely diagnosed in pediatric age [3]. 

Interestingly, children may present a mixed phenotype or even modify from a predominant phenotype to another during the course of disease and growth [4]. Children may remain asymptomatic for a long time or, with progression of disease, develop symptoms related to heart failure or arrhythmias, syncope or sudden cardiac death (SCD). Overall prognosis of CMPs in pediatric age is poor, and CMPs are considered responsible for approximately half of SCD or the need for cardiac transplantation occurring in childhood or adolescence [5]. Consequently, early detection of disease and comprehensive assessment of morphological alterations and risk factors is of paramount importance in order to initiate appropriate therapy and prevent complications, especially in young patients.

A multiparametric approach with integration of imaging and clinical data, electrocardiogram (ECG), familiar history and genetic analysis is crucial for complete assessment of CMPs. Non-invasive cardiac imaging techniques are fundamental tools to define the phenotypic and pathognomonic aspects of the various forms of CMPs.

Transthoracic echocardiography (TTE) is the standard imaging modality for diagnosis and follow-up of CMPs in children. TTE is a worldwide used, easily accessible, safe and low-cost technique that provides both morphological and functional data information. Technological advances, such as strain analysis and three-dimensional (3D) echocardiography, have recently improved its diagnostic and prognostic abilities. However, TTE is limited in its interobserver variability, acoustic window dependence and the absence of myocardial tissue characterization [6].

Performance of cardiovascular magnetic resonance (CMR) is of greatest value in the context of childhood CMPs secondary to its high diagnostic accuracy with excellent spatial and temporal resolution, lack of ionizing radiation, low-operator dependency and good reproducibility, which make it an ideal and unique technique for the assessment of congenital and acquired cardiac diseases in the pediatric age. CMR is considered the gold-standard for measurement of myocardial wall thickness, calculation of myocardial mass, heart chambers volumes and function, as well as detailed assessment of regional wall motion abnormalities. CMR also allows acquisition of 3D datasets of images and representation of cardiovascular anatomy, which is of fundamental value in children [7]. Moreover, CMR can provide in vivo tissue characterization and detect the presence of edema and fat in the myocardium, as well as pathological changes due to cardiac remodeling such as myocardial fibrosis, which may be evident at a young age [8]. Fibrosis may be present either with a localized, replacement pattern or diffuse pattern. Traditional late gadolinium enhancement (LGE) imaging assesses focal gross alterations of the myocardial structure with replacement fibrosis, but diffuse patterns of fibrosis may eventually be missed [9]. Therefore, novel parametric mapping sequences have become feasible in the last decade and are increasingly used as they can provide quantitative data on myocardial tissue with high sensitivity, in some cases also avoiding gadolinium-based contrast agent (GBCA) administration [10]. These sequences may add prognostic and therapeutic implications as demonstrated in recent studies [11,12].

In the last few years, cardiac computed tomography (CCT), which is a validated tool for the non-invasive assessment of coronary arteries, has also emerged as a valid alternative to CMR in the evaluation of CMPs, although its role in children is mainly limited by its radiation exposure. Novel applications of CCT for detection of myocardial fibrosis and volume quantification with latest generation scanners have shown good agreement with CMR data but are not currently used in clinical practice [13].

The aim of this review is to highlight the role of CMR in the management of the CMPs most frequently found in pediatric age, such as DCM, HCM and LVNC. This work focuses on the diagnostic and prognostic features of pediatric CMPs which can be assessed with CMR. Considerations about clinical issues, genetics and the spectrum of medical and surgical therapies are fundamental in the evaluation of pediatric CMPs but are beyond the scope of this paper. Integration of CMR in the preparation and follow-up phase of cardiac transplantation in children is also an issue, but evidence is still limited and it would be a very interesting topic to deal with in the next future.

## 2. Brief Technical Aspects

Recommendations for adequate performance of CMR studies in the pediatric population have been provided by recent consensus documents [14]. In the context of CMPs, the acquisition of sequences should be targeted principally on the assessment of ventricular volumes and ejection fraction, myocardial thickness, wall motion abnormalities, and tissue characterization.

For volumetric assessment and evaluation of kinetics, multiphase, possibly breath-holding, steady-state-free-precession (SSFP) cine sequences with retrospective ECG-gating should be acquired in short-axis and long-axis planes with a recommended slice thickness from 4 to 8 mm according to body weight. Reference values for children have been proposed [15].

For tissue characterization, sequences that are usually acquired include T1- and T2-weighted imaging, with and without fat saturation, T1-weighted sequences with and without GBCA and late gadolinium enhancement (LGE). Of note, the assessment of fibrosis with CMR is a cornerstone in the management of patients with CMPs and LGE represents the gold standard for the in vivo assessment of myocardial fibrotic tissue and showed an excellent correlation with histology [16]. Novel parametric sequences, such as native and post-contrast T1 and T2 mapping, have been increasingly used in clinical practice due to high diagnostic accuracy and prognostic power as demonstrated in studies concerning CMPs [17]. Such techniques allow a direct quantification of myocardial T1 and T2 relaxation times which are displayed on dedicated voxel-wise color-coded maps, in opposition to conventional T1- or T2-weighted imaging, which identify abnormal tissue alterations based on the relative image signal intensities of defined areas when compared to the normal myocardium. Parametric mapping can reveal the presence of subtle and diffuse myocardial alterations with higher sensitivity than classical sequences and at an earlier stage, when structural changes may still be reversible under treatment [18].

Complementary pure anatomical sequences, such as 3D balanced SSFP, may be added to exclude concomitant congenital vascular and thoracic anomalies. Of note, ECG and respiratory navigator-gated, free-breathing, 3D balanced SSFP sequences can achieve a 3D anatomic dataset with high spatial resolution (isotropic voxel size of 0.9–2 mm) and without the need for a contrast agent. In patients affected by HCM, stress protocols may be performed to assess patients for inducible myocardial ischemia, but few evidence exists on vasodilator perfusion imaging in children [19].

Sedation is generally required in CMR studies in children less than 5 years old. An optimal scanning setting, entertainment of children with audible books and films, and the presence of parents during the CMR examination can potentially avoid sedation at very young ages and improve image quality. In neonates, the “feed, swaddle, and sleep” technique can be applied with achievement of good scanning times [20]. Alternatively, deep sedation or general anesthesia can be used with a good safety profile and examination quality [21].

For tissue characterization and perfusion imaging, GBCA are commonly administered intravenously in pediatric patients of all ages. Many of them are often used “off-label” in this subset of patients, but the documented overall rate of adverse events in both adults and children is very low [22].

Several promising CMR techniques, including strain analysis, artificial intelligence and machine learning applications, have opened new frontiers for diagnosis and patient welfare in the last few years, although further validation is required to fully integrate them into routine clinical use [23].

## 3. Dilated Cardiomyopathy

DCM is currently defined by the presence of left ventricular (LV) or biventricular dilation and systolic dysfunction in the absence of other cardiac alterations such as coronary artery diseases, valvular, or congenital heart diseases [24]. Additional typical features of DCM are myocardial wall thinning due to progressive interstitial fibrosis, diffuse trabeculations and global hypokinesia [25]. Despite being the most common type of childhood CMP, it is a rare disorder, with an overall annual incidence of approximately 0.58–0.7 per 100,000 children, which becomes highest during the first year of life [26]. In most cases the etiology remains unknown (“idiopathic” DCM), but genetic tests have recently shown that approximately 40% of cases are familial, with mutations involving predominantly sarcomeric proteins [27]. Other forms of DCM in children could be manifestations of infectious diseases (particularly viral myocarditis), autoimmune and systemic disorders, toxic insults (including chemotherapy), metabolic and nutritional alterations (lysosomal storage diseases, carnitine deficiencies) and neuromuscular diseases (such as Duchenne/Becker muscular dystrophies) [28].

Clinically, signs of congestive heart failure are described in 70–90% of children, with the most severe forms during the first year of life [29]. Despite recent advances in pharmacological and surgical treatment, data from pediatric registries show a transplant-free survival of approximately half of the cases after 20 years and DCM is currently the most common indication for heart transplantation in the pediatric age [30]. Presence of severe systolic impairment, younger age, history of familial DCM, and absence of improvement of cardiac function despite optimal medical therapy at follow-up are considered major risk factors for poor prognosis [29].

### CMR Diagnostic and Prognostic Role in Pediatric DCM

Among all imaging modalities, TTE represents the standard diagnostic approach in children with DCM, but in recent years CMR has shown great applicability, diagnostic and prognostic power in pediatric patients with DCM [31]. Use of CCT is rare, and generally targeted at accurately defining coronary arteries’ origin, course, and termination. Phase contrast CMR is a useful alternative to echocardiography for evaluating severity of functional mitral regurgitation that may develop as a consequence of LV dilatation. Assessment of tissue characterization by CMR can define the presence, extent and distribution of myocardial damage.

In neonates with development of severe LV dilatation and dysfunction, a complete diagnostic approach should be addressed in order to exclude presence of ongoing myocarditis, or, most probably, of congenital coronary artery anomalies. In this case, TTE may reveal anomalous origin or proximal course of epicardial coronary arteries in 90% of cases and detect regional LV wall motion abnormalities. However, in the most challenging cases invasive coronary angiography may be performed to confirm the diagnosis. Among second-level non-invasive imaging techniques, in neonates with a very high heart rate CCT should be preferred, while in older children CMR angiography can be performed with high diagnostic accuracy and no radiation exposure [32]. LGE may often detect signs of myocardial infarction as a consequence of coronary anomalies, which is typically subendocardial with various degrees of transmurality and involving segments supplied by specific coronaries [33]. On the other hand, myocarditis typically presents positive LGE with non-ischemic, subepicardial or mid-wall LGE in the inferolateral wall or anterior septum. Concomitant edema or hyperemia may also be evident. Acute myocarditis is a relatively frequent cause of DCM among children, being confirmed in about one third of early endomyocardial biopsies [34].

In young patients affected by Duchenne muscular dystrophy (DMD) cardiac involvement with CMP represents the major cause of death [35]. Typical early cardiac findings in DMD are myocardial edema and subepicardial fibrosis of the LV, predominantly in the basal inferolateral wall, with preserved ejection fraction, but at a later stage in 7–18% of patients DCM may develop [36,37].

**Figure 1:**
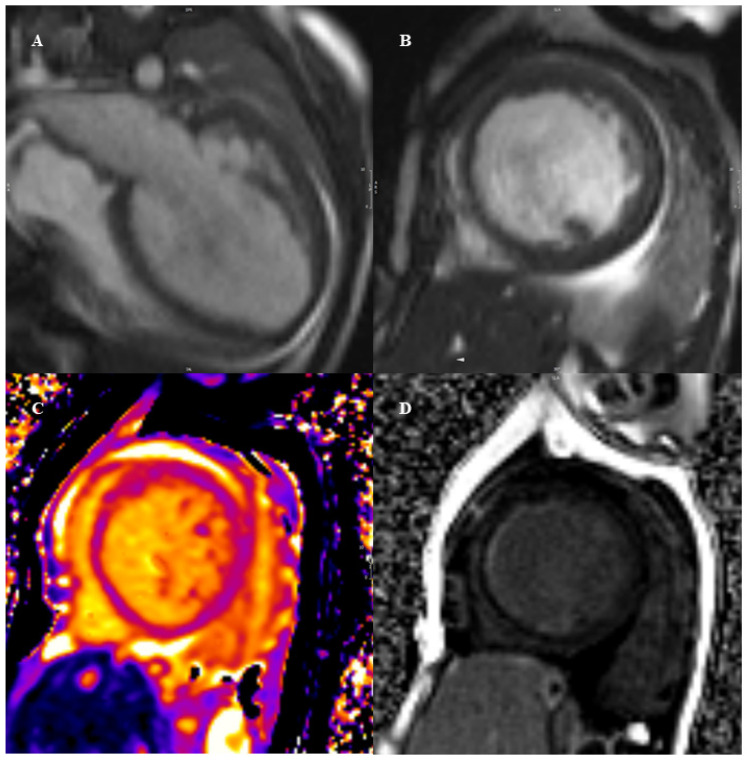
A case of DCM in a 6-year-old child, male. Cine sequences documented diffuse hypokinesia, septal dyssynergy and severe left ventricular dilatation (left ventricular end diastolic volume indexed 158 mL/m^2^) with a severely compromised ejection fraction (LVEF 12%) as shown in panels (**A**,**B**). The T2-mapping sequences showed diffusely elevated T2 values (72–75 ms), as a sign of concomitant myocardial edema (panel (**C**)), which was not evident in T2-weighted imaging. Late gadolinium enhancement was suggestive for septal mid-wall hyperenhancement (panel (**D**)).

Advanced forms of DCM may show presence of characteristic linear mid-wall LGE in a non-coronary distribution, usually in the interventricular septum (“mid-wall fibrosis”), which is reported in approximately 30% of DCM cases of all ages [37], and correlates with a poor prognosis [11,38]. Of note, distribution pattern of LGE in children may be more heterogeneous than in the adult population. A recent retrospective study by Muscogiuri et al. described a characteristic global, diffuse, subendocardial LGE involving papillary muscles and trabeculae in 15 children affected by DCM, which was related to reduced LVEF and global longitudinal strain [39]. In a previous single-center study in children with DCM and congestive heart failure, LGE was reported in only 16% of patients, including mid-wall, focal, patchy, right ventricle insertion sites and transmural types [40]. Based on these results, extreme variability exists in the LGE presentation form of DCM among young patients and should be further investigated. An example of a pediatric DCM in CMR is shown in Figure 1.

DCM is also associated with an increased risk of ventricular arrhythmias and SCD. Risk stratification and indication for ICD implantation in primary prevention in children affected by DCM is extremely challenging due to the lack of randomized, multicenter studies in the pediatric population. LVEF has historically been considered the most relevant factor for risk stratification, but presence of LGE was revealed as a powerful predictor of fatal arrhythmias and SCD in many studies [41]. In young carriers of lamin A/C mutations, mid-wall fibrosis was present in 88% of cases, and was significantly correlated with conduction abnormalities [42]. Positive LGE also characterizes patients with reduced likelihood of reverse cardiac remodeling, which is the normalization of cardiac chamber volumes and function under pharmacological therapy [43]. Both native T1 and ECV mapping parameters seem to significantly affect prognosis in adults with DCM, independently of both LVEF and LGE and high native T1 values seem to represent early markers of adverse remodeling before the development of LGE [44]. A recent single-center study however outlined how there was no significant difference in survival between children with higher versus lower native T1 values [45].

Table 1 shows the recommended CMR protocol with standard and additional sequences and typical findings for the assessment of DCM among children. Diagnosis of DCM in children with CMR is mainly achieved with standard 2D cine sequences but, differently from the adult population, less uniformity exists in terms of tissue characterization. In children, LGE and parametric mapping patterns are very variable and not highly specific for disease.

DCM presents an extremely bad prognosis in neonatal age, and CMR data concerning follow-up of the cardiomyopathy are poor, as many of these children die or undergo cardiac transplantation in the first months of life. Moreover, prognostic data provided by CMR are still limited to small, single-center studies and larger, prospective, randomized trials are needed for further validation.

## 4. Hypertrophic Cardiomyopathy

HCM is characterized by excessive and inappropriate thickening of the ventricular myocardium in the absence of loading conditions responsible for it. Other typical morphological features of HCM are myocardial crypts, hypertrophic and apically displaced papillary muscles, and elongated leaflets with systolic anterior motion (SAM) of the mitral valve (MV) in obstructive forms of HCM [46,47]. In children, HCM is the second most common group of CMPs, covering 25–42% of all cases. It shows a prevalence of 1:500 in the adult population and an annual incidence of 0.24–0.47 per 100,000 children [26]. It is considered a genetic disorder with prevalent familial origin and autosomal dominant inheritance. Sarcomeric protein mutations, especially those of β-myosin heavy chain genes, such as MYBPC3 or MYH7, are documented in 60% of all pediatric cases [48].

In childhood, HCM presentation could be more heterogeneous and in some cases related to specific malformation syndromes, metabolic defects or neuromuscular conditions, such as Pompe disease, Noonan syndrome and Friedreich ataxia [49]. Clinical presentation may range from asymptomatic to symptoms such as exercise intolerance, chest pain, syncope, or cardiac arrest, which is an uncommon early presentation in childhood [50]. The highest risk of mortality is found in those with a very early diagnosis in the first year of life [51]. Relevant risk factors for worse outcomes among children are symptomatic forms, massive and concentric LV hypertrophy with restrictive pattern at initial diagnosis, Noonan syndrome, and progressive forms with development of LV systolic dysfunction [52].

### CMR Diagnostic and Prognostic Role in Pediatric HCM

Diagnosis of HCM requires exclusion of other causes of LV hypertrophy, similar to HCM but with a different etiology, which are known as HCM phenocopies. In children and young adults, most common HCM phenocopies are physiologic remodeling secondary to long-standing training (athlete’s heart), metabolic and storage diseases, and cardiac tumors [53]. Consequently, non-invasive imaging techniques are fundamental for correct patient management.

TTE is currently the mainstay for standard diagnostic approach and follow-up, as well as for familial screening. In adults, diagnosis of HCM can be made when the maximal end-diastolic wall thickness is ≥15 mm in one or more myocardial segments, in the absence of other causes of LV hypertrophy. In children, LV hypertrophy is considered when LV wall thickness is ≥2 standard deviations above the mean (z score ≥ 2) for age, sex and body size [46].

The classical phenotype of HCM is characterized by asymmetric hypertrophy, prevalent at the basal anterior septum (70% of all patients), while less common types are non-septal asymmetric hypertrophy, symmetric and concentric hypertrophy, right ventricular (RV) and apical HCM [54]. Basal septal hypertrophy can lead to LV outflow tract (LVOT) obstruction due to a combined mechanism of basal septum hypertrophy and SAM of the MV [55]. Diastolic dysfunction with left atrium (LA) dilatation is seen due to the massive muscular mass and stiffness, while only late forms present with severe dilatation and systolic impairment (“burned-out” or end-stage phenotypes). However, the only important parameter for diagnosis is given by maximal wall thickness, which may be challenging to be assessed with echocardiography [56].

Second-level imaging, principally with CMR, is often needed for diagnostic confirmation. CMR has emerged as a powerful diagnostic tool able to refine these challenging diagnoses, given its ability to define the extension and localization of hypertrophy and its ability in soft tissue characterization [57]. A recent consensus paper by the Society for Cardiovascular Magnetic Resonance (SCMR), recommends CMR in all patients with HCM [58]. HCM typically presents a non-ischemic, mid-wall LGE with a patchy distribution and does not follow a coronary artery distribution, prevalent in the hypertrophied areas and at the RV insertion points in the interventricular septum [59].

Positive LGE is described in approximately 60% of adult patients with HCM, although its real prevalence in children and adolescents is not well defined. A recent study by Raja et al. described a 46% prevalence of LGE in children with overt HCM [60]. In contrast to adult patients, LGE distribution pattern is less defined in children. A study by Windram et al. described a prevalent septal involvement of LGE, with a lower percentage than in adults [61]. Moreover, in young patients after septal ablation procedures, LGE can show the exact localization and extension of myocardial damage [62].

**Figure 2:**
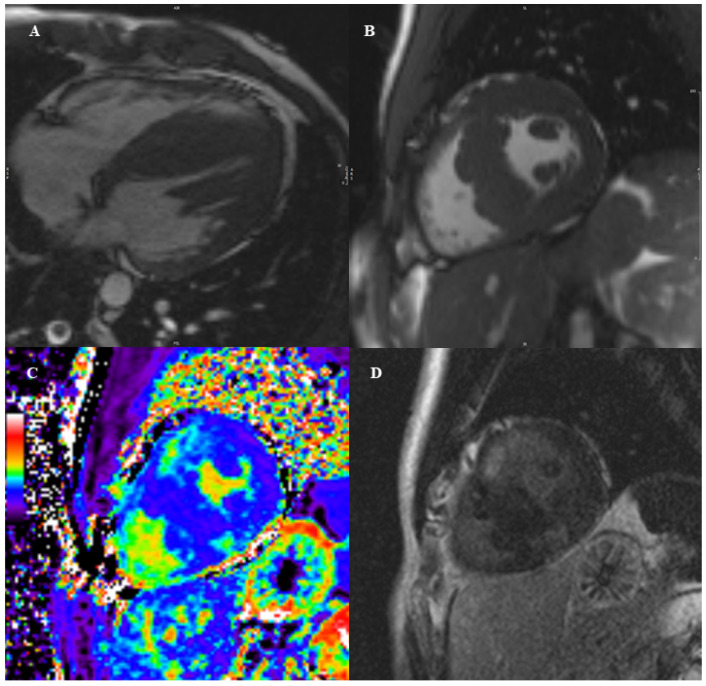
13 year-old boy with positive family history for HCM. The CMR examination showed an asymmetric hypertrophy with prevalent involvement of the interventricular septum with a maximal wall thickness of 34 mm, and hypertrophy of papillary muscles (panel (**A**,**B**)), which was highly suggestive for HCM. To implement diagnosis and risk stratification, we then performed tissue characterization sequences. We added parametric mapping sequences before and after contrast injection. As shown in panel (**C**), the calculated extracellular volume in the mid anterior and posterior septal wall was elevated (38%), as a sign of diffuse fibrosis. In the same segments late gadolinium enhancement was also evident with a patchy, intramyocardial distribution, as a sign of replacement fibrosis (panel (**D**).

In pediatric patients, diffusely increased T1 values and decreased global longitudinal strain values were found in HCM patients compared to controls [63], as a sign of global involvement of the myocardium. Regarding ECV values, which reflect the presence of fibrosis in the interstitial space, a recent CMR study outlined how septal ECV values were not significantly increased after surgical septal myectomy when performed in early childhood in HCM with LVOT obstruction resistant to medical therapy [64]. LA strain assessed by CMR in pediatric HCM patients with normal LA volumes were closely linked to the amount of total LV LGE and presence of diastolic dysfunction and may represent a useful early marker in children to detect initial structural and functional changes [65]. A representative case of pediatric HCM is demonstrated in Figure 2.

HCM is the most common cause of SCD in young patients, and a complete identification of specific pediatric risk factors for SCD is fundamental to guiding implantable cardioverter defibrillator (ICD) implantation. If an indication for implantation of ICD in secondary prevention is well established, in the context of primary prevention from SCD, the selection of candidates for ICD implantation is often challenging and multiple SCD risk factors have been developed in the last few years. In a recent systematic review, a history of previous adverse cardiac events, non-sustained ventricular tachycardia, unexplained syncope, and very severe LV hypertrophy have been identified as major risk factors for SCD in children with HCM [66]. Norrish et al. formulated a novel risk prediction model for SCD in childhood HCM, which included measurement of maximal left ventricular wall thickness and LA diameter together with clinical characteristics [67]. Another SCD risk prediction model for children was presented by Miron et al., who added the age of disease presentation and the presence of a pathogenic gene mutation [68]. However, these risk scores include only some indicators of global arrhythmic risk. In pediatric HCM, differently from adults, there is a non-linear correlation of the degree of hypertrophy and SCD risk so that no cut-off of myocardial thickness z-score can be identified to recommend ICD implantation [68,69]. HCM phenotypes with apical aneurysm are at high risk for SCD and thromboembolic complications, but evidence is currently still limited to a series of selected adult patients [70].

The presence and extent of LGE have shown important prognostic implications in both adults and children with HCM, as extensive fibrosis is associated with a higher risk for later LV dilatation and systolic dysfunction [22,71]. In children, LGE was found to be a strong predictor of worsened diastolic dysfunction, major arrhythmias, SCD, or appropriate ICD intervention, independent from LVEF [72]. A cut-off of LGE >15% of total left ventricular myocardial mass has been proposed by Mentias et al. in a recent study to identify adult patients at higher risk of SCD [73]. Positive LGE could therefore indicate the need for ICD implantation, although there is no conclusive evidence [74]. A potential prognostic role of mapping values has been underlined in recent studies [74]. A recommended CMR protocol for pediatric HCM and its typical features is represented in Table 2.

Comprehensively, the diagnosis of HCM is provided by 2D cine SSFP sequences supported by positive LGE in most hypertrophied segments. The presence of LGE is less frequent than in adults but typically presents with an intramyocardial, patchy pattern of distribution. In contrast, risk stratification of children with HCM is still challenging, and no uniformity exists on clinical risk scores and prognostic data, which require more detailed investigation and standardization.

## 5. Left Ventricular Non-Compaction

LVNC is a heterogenous type of CMP characterized by excessively elongated ventricular trabeculations separated by wide and deep intertrabecular recesses communicating with the ventricular cavity, usually associated with a thinner compacted layer [75]. Other typical features of LVNC are LV systolic and diastolic dysfunction, intraventricular thrombi, abnormal papillary muscles, and segmental distribution of non-compacted regions, affecting predominantly the middle and distal parts of the inferior and lateral walls and with no involvement of the interventricular septum [76]. Prevalence ranges from <0.3% to 1.26% in young children, while in retrospective studies, authors documented a prevalence of approximately 9% in children affected by other CMPs [77].

Currently, there is still much debate about whether to consider LVNC as a distinct entity or a morphological variant phenotype of LV hypertrabeculation. On one side, the ESC and the World Health Organization include LVNC in the group of unclassified CMPs, while the American College of Cardiology/American Heart Association (ACC/AHA) classifies LVNC as a distinct primary genetic cardiomyopathy [78,79].

Comprehensively, such disease may be isolated or associated with other CMPs, congenital heart diseases, or specific syndromes. For example, LVNC has been reported in patients affected by Ebstein anomaly (25% of all cases), Barth syndrome, metabolic myopathies, mitochondrial disorders, neuromuscular diseases, and genetic syndromes. The most common genetic defects involve sarcomeric proteins [80]. Many genetic mutations have shown a large overlap with other CMPs and congenital cardiac defects and many LVNC patients may present morphological aspects of DCM, and occasionally of HCM (in particular the apical variant) [81].

### CMR Diagnostic and Prognostic Role in Pediatric HCM

Imaging techniques allow diagnosis of LVNC, but there is no formal consensus for diagnostic criteria which are still debated. This causes great uncertainty not only related to the presence or absence of the disease, but also in defining its real prevalence, etiology, therapy and prognosis and results in a cardiac imaging challenge [82]. LV hypertrabeculations may also be present in healthy subjects, and it may not be easy to distinguish these from early stages of pathological LVNC [83]. Moreover, it may be particularly difficult to differentiate LVNC from DCM in children, since the presence of LV dilatation and systolic dysfunction has also been described in cases of LVNC without primary DCM [84]. The first-line diagnostic modality is TTE, but in the last few years there has been a wider application of CMR and even CCT. Assessment of LVNC by TTE is based principally on three groups of diagnostic criteria, but no standard agreement exists and these were derived only from small groups of patient datasets [85].

CMR offers unlimited views to all cardiac structures with high spatial resolution, which is particularly useful in cases of suspected LVNC due to excellent visualization of all apical segments, which are typically involved in non-compaction. As for TTE, different sets of diagnostic criteria by CMR have been proposed. First in 2005, Petersen et al. proposed a ratio of non-compacted layer to compacted layer (NCL/CL) ≥2.3 measured in a long-axis view at end-diastole as diagnostic for LVNC. This showed a sensitivity of 86% and specificity of 99% in patients with a known LVNC diagnosis [86]. Later, in 2010, the method by Jacquier et al. diagnosed LVNC when the non-compacted mass measured in short-axis view at end-diastole was greater than 20% of the total mass. In this prospective study, 16 LVNC patients were recruited and showed a higher sensitivity of 94% with a specificity of 94.5% [87]. In 2012, the group of Grothoff et al. proposed four criteria based on non-compacted mass, NCL/CL ratio and its distribution to define LVNC. This method showed a sensitivity of 75% and a specificity of 100% in diagnosing LVNC [88]. In 2013, Stacey et al. considered a cut-off of NCL/CL ratio of 2 to define the presence of LVNC when measured in short-axis view at end-systole [89]. In 2016 Choi et al. documented a sensitivity of 66% and a specificity of 90% proposing a trabecular mass greater than 35% as diagnostic for LVNC [90]. Captur et al. found that LVNC patients had a global LV fractal dimension (FD) higher than normal subjects due to the extensive trabecular component and introduced it as a diagnostic criteria [91].

**Figure 3:**
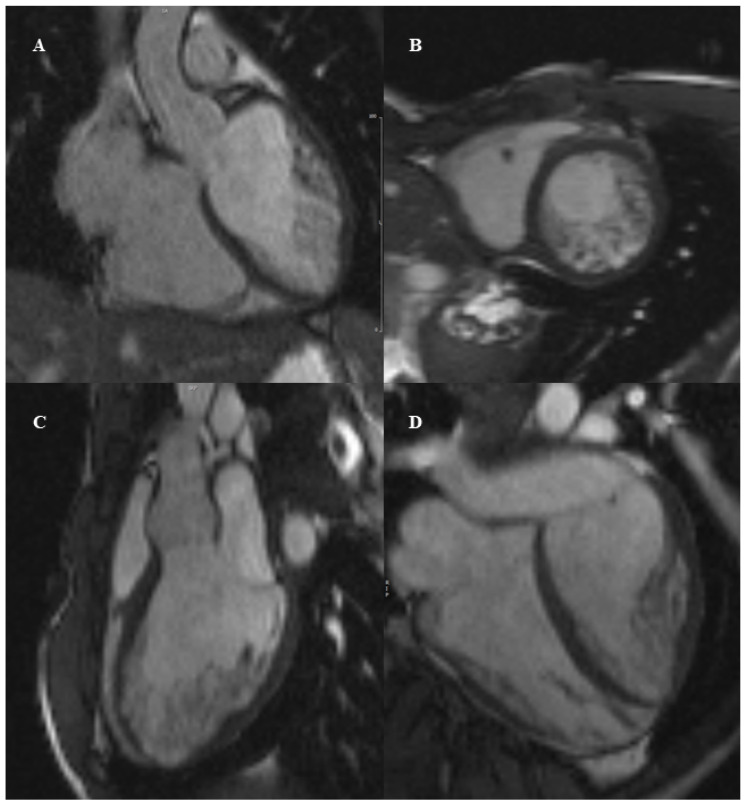
Twelve year-old child, male. Cine sequences were performed in the long-axis (panel **A**,**C**,**D**) and short-axis view (panel **B**) to assess for left ventricular non compaction. The measured non-compacted/compacted ratio was 3.4–4.7 with prevalent hypertrabeculations at the lateral wall, while the septum appears spared. Overall left ventricular function was only mild decreased (ejection fraction 47%).

However, all these methods were derived from small population samples and no formal agreement exists about their use for diagnosis of LVNC. In the Screen to Prevent (S2P) Study, a cross-sectional study involving 5169 children in Texas, CMR was used as a screening modality to identify congenital cardiac anomalies associated with SCD. LVNC was incidentally detected by CMR in 959 cases (18.6%), according to the Petersen criteria [92]. The same authors then reexamined the CMR data of LVNC patients detected in the S2P study and found different prevalence of 17.5%, 7.4% and 1.3% of LVNC by applying the Jacquier criterion, the Choi criterion and the Grothoff criterion, respectively. Although the study was limited by the absence of longitudinal data, it underscored the lack of concordance among all criteria [93]. Diagnosis of LVNC by CMR requires cine-sequences and no tissue characterization is listed to achieve diagnosis. LGE has shown very low specificity and no distinct pattern for LVNC; eventually, contrast sequences may detect the presence of intertrabecular thrombi with high sensitivity and specificity [94]. Figure 3 represents a case of LVNC detected by CMR in a young boy.

An alternative technique for diagnosis as well as for follow-up of LVNC is CCT, although it is not routinely used for children due to its radiation exposure. A cut-off value of NCL/CL greater than 2.2 or even 1.8 has been proposed in recent studies [95], but no consensus criteria exist for LVNC diagnosis by CCT.

The long-term outcome of LVNC is highly variable and related to the age of presentation and the phenotype manifestation. Early presentation in the pediatric age, especially in the first year of life, is recognized as an independent risk factor and associated with ventricular arrythmias and SCD [96]. In addition to clinical aspects, cardiac imaging modalities can provide important insights into LVNC prognosis and contribute to patients’ risk assessment. Recent reviews found the highest risk of mortality and SCD in the dilated and mixed or indeterminate phenotype, followed by the hypertrophic one [4]. LV systolic dysfunction has also been related to poor outcomes [97] and may develop due to the fact that the prevalent contribution to systolic contraction is given by the subendocardial layers, which are typically altered in LVNC. Additionally, reduced myocardial perfusion in the non-compacted layers has been demonstrated in CMR studies [98]. Other negative predictive features are an extended disease with involvement of the posterior wall, and presence and extent of focal and diffuse myocardial fibrosis assessed with LGE and T1 mapping, respectively [99].

Given the lack of definitive diagnostic criteria, in the presence of extensive trabeculation, follow-up with echocardiography and eventually CMR should be regularly performed even in asymptomatic children to assess for potential remodeling, intraventricular thrombi and systolic dysfunction. Ivanov et al. assessed LVNC prevalence by 4 sets of criteria and adverse events at 7-year-follow-up in a large adult population of non-selected patients referred for CMR. Overall prevalence of LVNC was high (39% by Petersen, 23% by Stacey, 25% by Jacquier and 3% by Captur criteria) and no significant correlation was found between presence of LVNC and adverse clinical outcome, independent of the applied criteria. Moreover, an NCL/CL ratio > 3 as assessed with Petersen criteria did not show any correlation with future cardiovascular events [100].

Standard and additional CMR sequences for a complete assessment of LVNC cardiomyopathy in children is described in Table 3.

Given the high prevalence of disease, a simplified diagnostic path should be provided for the diagnosis of LVNC. Current evidence is based on a paucity of data and many parameters result often discordant and not always feasible in the same patients (for example fractal analysis). Concerning prognosis, CMR data are still limited and a comprehensive assessment of concomitant systolic dysfunction, arrhythmias and positive family history is of paramount importance for risk stratification and follow-up.

## 6. Conclusions

Although echocardiography remains the standard frontline diagnostic approach and is an ideal tool for periodic re-evaluation of childhood CMPs, growing evidence supports the integration of CMR into routine clinical practice. CMR can be safely performed in the pediatric population and, in addition to echocardiography, can provide information on tissue characterization. The presence and distribution of fibrosis aids the recognition of CMPs from differential diagnoses. However, differently from the adult population, findings in children are more variable and less specific. Concerning risk stratification and follow-up, data in children are lacking as prognosis is poor and the main studies are restricted to single research groups. Given the potential value of CMR in terms of diagnosis and risk assessment in childhood CMPs, a further standardized prospective investigation in larger pediatric populations is warranted.

## Figures and Tables

**Table 1:** Standard and additional CMR sequences for assessment of DCM in children.

Standard CMR Sequences	Typical Findings
Localizer (*transaxial*, *coronal*, *sagittal*)	
2D Cine bSSFP	▪ ventricular dilatation and impairment of systolic function ▪ global hypokinesia
Late Gadolinium Enhancement	▪ very variable: absent, intramyocardial, transmural, subendocardial or subepicardial; septal linear mid-wall sign
**Additional CMR Sequences**	
Native T1 and ECV mapping	▪ normal or global increase of native T1 and ECV values
T2 mapping and T2-weighted (STIR)	▪ normal or global increase of native T2 values and T2 signal intensity (acute phase of decompensation)
2D velocity-encoded phase contrast	▪ assessment of mitral valve regurgitation secondary to annular dilatation ▪ assessment of aortic flow for cardiac index calculation
3D whole-heart bSSFP	▪ to exclude anomalies of origin/course/termination of the coronary arteries

CMR cardiovascular magnetic resonance; DCM dilated cardiomyopathy; 2D two-dimensional; bSSFP balanced steady state free precession; ECV extracellular volume; 3D three-dimensional; STIR short-tau inversion recovery.

**Table 2:** Standard and additional CMR sequences for assessment of HCM in children.

Standard CMR Sequences	Typical Findings
**Localizer** (*transaxial*, *coronal*, *sagittal*)	
**2D Cine bSSFP**	▪ increased myocardial wall thickness ≥ 2 standard deviations above the mean (z score ≥ 2) for age, sex and body size ▪ small ventricular cavities with hypercontractile function ▪ mainly asymmetric hypertrophy with septal involvement, but also mid-ventricular, apical, concentric or right ventricular hypertrophy ▪ myocardial crypts, hypertrophied and apically displaced papillary muscles, elongated mitral valve leaflets, apical aneurysm ▪ obstructive forms with outflow tract obstruction due to septal hypertrophy and systolic anterior motion of the mitral valve or intracavitary obstruction due to mid-ventricular hypertrophy
**Late Gadolinium Enhancement**	▪ patchy, intramyocardial distribution in most hypertrophied segments
**Additional CMR Sequences**	
Native T1 and ECV mapping	▪ regional increase of native T1 and ECV values in most hypertrophied segments
2D velocity-encoded phase contrast	▪ assessment of mitral valve regurgitation due to SAM

CMR cardiovascular magnetic resonance; HCM hypertrophic cardiomyopathy; 2D two-dimensional; bSSFP balanced steady state free precession; RV right ventricle; ECV extracellular volume.

**Table 3:** Standard and additional CMR sequences for assessment of LVNC in children.

Standard CMR Sequences	Typical Findings
**Localizer** (*transaxial*, *coronal*, *sagittal*)	
**2D Cine bSSFP**	▪ assessment of LV systolic function (preserved or reduced) ▪ end diastolic ratio NC/C ≥ 2.3 (Petersen criteria) ▪ end diastolic ratio NC mass > 20% total left ventricular mass (Jacquier criteria) ▪ end systolic ratio NC/C ≥ 2.0 (Stacey criteria) ▪ Fractal analysis (Capture criteria), seldom used
**Additional CMR Sequences**	
Native T1 and ECV mapping	▪ normal or global increase of native T1 and ECV values
Late Gadolinium Enhancement	▪ very variable: absent, intramyocardial, subendocardial, subepicardial, transmural; in severe forms fibrosis of spongiform myocardium possible

CMR cardiovascular magnetic resonance; LVNC left ventricular non compaction; 2D two-dimensional; bSSFP balanced steady state free precession; LV left ventricular; NC/C non compacted to compacted ratio; 3D three-dimensional; ECV extracellular volume.
